# Fabrication and Characterization of PCL/PLGA Coaxial and Bilayer Fibrous Scaffolds for Tissue Engineering

**DOI:** 10.3390/ma14216295

**Published:** 2021-10-22

**Authors:** Morteza Bazgir, Wei Zhang, Ximu Zhang, Jacobo Elies, Morvarid Saeinasab, Phil Coates, Mansour Youseffi, Farshid Sefat

**Affiliations:** 1 Department of Biomedical and Electronics Engineering, School of Engineering, University of Bradford, Bradford BD7 1DP, UK; m.bazgir@student.bradford.ac.uk (M.B.); m.youseffi@bradford.ac.uk (M.Y.); 2 State Key Laboratory of Polymer Materials Engineering, Polymer Research Institute at Sichuan University, Chengdu 610065, China; weizhang@scu.edu.cn; 3 Advanced Polymer Materials Research Center, Sichuan University, Shishi 362700, China; 4 Chongqing Key Laboratory of Oral Disease and Biomedical Sciences and Chongqing Municipal Key Laboratory of Oral Biomedical Engineering, Higher Education and Stomatological Hospital, Chongqing Medical University, Chongqing 401174, China; zhangximu@hospital.cqmu.edu.cn; 5 School of Pharmacy and Medical Sciences, Faculty of Life Sciences, University of Bradford, Bradford BD7 1DP, UK; j.eliesgomez@bradford.ac.uk; 6 Department of Biology, Faculty of Science, Ferdowsi University of Mashhad, Mashhad 91779-4897, Iran; m.saeinasab@gmail.com; 7 Interdisciplinary Research Centre in Polymer Science and Technology (Polymer IRC), University of Bradford, Bradford BD7 1DP, UK; P.D.Coates@bradford.ac.uk

**Keywords:** electrospinning, coaxial, bilayer, polycaprolactone (PCL), poly lactic-co-glycolic acid (PLGA), contact angle, mechanical properties, degradation

## Abstract

Electrospinning is an innovative new fibre technology that aims to design and fabricate membranes suitable for a wide range of tissue engineering (TE) applications including vascular grafts, which is the main objective of this research work. This study dealt with fabricating and characterising bilayer structures comprised of an electrospun sheet made of polycaprolactone (PCL, inner layer) and an outer layer made of poly lactic-co-glycolic acid (PLGA) and a coaxial porous scaffold with a micrometre fibre structure was successfully produced. The membranes’ propriety for intended biomedical applications was assessed by evaluating their morphological structure/physical properties and structural integrity when they underwent the degradation process. A scanning electron microscope (SEM) was used to assess changes in the electrospun scaffolds’ structural morphology such as in their fibre diameter, pore size (μm) and the porosity of the scaffold surface which was measured with Image J software. During the 12-week degradation process at room temperature, most of the scaffolds showed a similar trend in their degradation rate except the 60 min scaffolds. The coaxial scaffold had significantly less mass loss than the bilayer PCL/PLGA scaffold with 1.348% and 18.3%, respectively. The mechanical properties of the fibrous membranes were measured and the coaxial scaffolds showed greater tensile strength and elongation at break (%) compared to the bilayer scaffolds. According to the results obtained in this study, it can be concluded that a scaffold made with a coaxial needle is more suitable for tissue engineering applications due to the improved quality and functionality of the resulting polymeric membrane compared to the basic electrospinning process. However, whilst fabricating a vascular graft is the main aim of this research work, the biological data will not present in this paper.

## 1. Introduction

The goal of tissue engineering is to allow human cells to replace the implanted scaffold over time. Therefore, the scaffold must be biodegradable in order for cells to produce their extracellular matrix. The by-products of this decomposition must also be non-toxic and able to leave the body without disrupting the functioning of other organs [1]. One of the most potent preconditions for evaluating the effectiveness of a polymer processing system is understanding the degradation/corrosion of the scaffold in a biological environment [2]. Degradable polymers can be divided into two categories according to their biodegradability methods: heterogeneous (surface) and homogeneous (bulk) [3]. With surface biodegradation, polymer erosion is limited by the surface of the material, so the material loses its thickness over time while maintaining its structural integrity at all stages of deterioration [4,5]. Surface corrosion occurs when the rate of bond breaking (hydrolysis) is higher than the rate of water diffusion into the polymer [6]. In contrast to surface corrosion, when the water penetration rate into the material exceeds the rate of polymer hydrolysis, overall corrosion occurs. As a result, the corrosion of the entire material causes a sudden and rapid loss of structural integrity and mechanical strength, as well as changes in molecular weight and an increase in water content in the polymer, followed by the release of soluble monomers and chain components [4,7].

In a biological environment, polymers can be chemically degraded by hydrolysis or enzymatic degradation. Synthetic polymers (such as polyesters) are further degraded by hydrolysis, while enzymatic reaction usually degrades biopolymers [3]. Hydrolysis polymer decomposition is the result of many events. First, the water enters the polymer structure and causes swelling, and then the water molecules split covalent bonds between repeating units to form oligomers which causes the polymeric structure to become uneven and porous [8,9,10]. In polymer structures, the polymer chain can be crystalline or amorphous. The amorphous structure consists of polymer chains with random spacing, whereas the crystal structure is very regular similarly to the arrangement of structures within a crystal structure [11,12]. The semi-crystalline structure consists of these two structural regions and has been shown to be useful in the tissue engineering field [13,14].

The electrospinning process is considered the easiest, most versatile and cost-effective way of producing nonwoven fibrous porous structures [15,16]. Nanofibres can be obtained both from a polymer melt or from a solution. In the field of tissue engineering, most of the scaffolds fabricated by the electrospinning process were solution-based. Various solutions, including natural and synthetic polymers and polymer blends have been electrospun into interconnected fibre structures with diameters ranging from nanometres to micrometres [17,18,19]. Some synthetic polymers lack some preferred properties in tissue engineering, such as being hydrophilic, promoting cell adhesion; however, some of these polymers have adequate and robust mechanical properties [20]. To overcome these problems is preferable to use two or more other polymers to make a porous scaffold [21,22].

**Figure 1:**
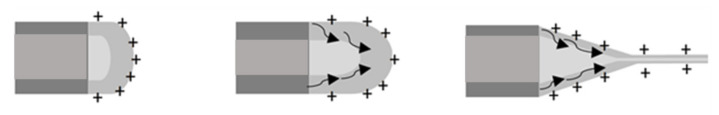
Schematic illustration of the Taylor cone formation of the polymeric solution at the tip of the coaxial needle, where the core (inner needle) is PCL and the shell is PLGA solution.

Aside from blending the polymer to obtain composite polymers into single nanofibres, another form of composite nanofibres can be obtained as the core and the shell of fibre to be entirely made of different types of polymeric solutions. This kind of fibres structure can be produced with a specialized needle known as the coaxial needle; Figure 1 below shows a simple schematic illustration of events that would occur at the tip of a coaxial needle during electrospinning. Loscertales et al. were the first to demonstrate the possibility of electrospraying two separate liquid solutions by the coaxial needle (the inner “core” liquid was coloured ethylene glycol (EG), and the outer solution was DuPont photopolymer Somos^®^ 6120); a year later, the method was further developed by Sun et al., 2003 who used this process to produce nanofibres which the core and the shell of the fibres made by the same 3 wt% poly(ethylene oxide) solutions [23,24]. Since then, coaxial electrospinning has become very popular in the biomedical/regenerative medicine and the pharmaceutical industries [25,26,27]. Coaxial electrospinning depends on the same process parameters as the single nozzle electrospinning, and it is arguably one of the cheapest and most efficient ways to produce microscopic diameter biopolymer composite fibres [28,29,30]. The fibre core material mainly determines the mechanical properties, and the polymer shell provides functions or properties such as cell adhesion and hydrophobicity/hydrophilicity [31,32,33].

Additionally, fabricating bilayer or multi-layers of the electrospun membrane can be useful for designing a material that will traverse multiple cell types to enhance cell infiltration and vascularization for tissue regeneration applications [34,35]. This method has the potential to develop polymer scaffolds with different degradation and mechanical characteristics that can be used as functional devices that will accurately mimic the complex natural components of the extracellular matrix. The novel bilayer electrospun biodegradable polymers have been evaluated in several studies as potential scaffolds for wound dressing, skin and bone regeneration, as well as artificial blood vessels [36,37,38,39,40,41]. Although multi-layer electrospinning has excellent biomedical potential, it is worth noting that the potential problems include selecting suitable biodegradable polymers with controlled porosity and wettability, choice of cell culture conditions, and the optimization of cell seeding methods should be solved to avoid in vivo implantation failure [19,42,43]. In this study, both methods were used to produce scaffolds from PCL and PLGA.

## 2. Experimental

### 2.1. Materials

PURASORB poly (lactic-co-glycolic acid) 82:18 was obtained from Corbion Netherlands and Poly (ε-caprolactone) with an average molecular weight wt. Mn 80,000 and density of 1.145 g/mL at room temperature, purchased from Sigma-Aldrich, UK. N, N-dimethylformamide (DMF), tetrahydrofuran (THF) and chloroform (CF), supplied by Fisher Scientific, UK, without prior purification, were used as solvents.

### 2.2. Solution Preparation and Electrospinning Parameter

The core and shell solutions used for coaxial electrospinning were PCL (15 wt%) and PLGA (10 wt%), respectively. With the same batch of polymeric solutions, the bilayer scaffolds were fabricated, where the inner layer is PCL, and the outer layer is made of PLGA nanofibres. The polymeric solutions were prepared by dissolving 9 g of PCL pellets in 51 g of chloroform, 6 g of PLGA in 27 g of THF and 27 g of DMF (50:50). The Solutions were placed on a magnetic stirrer in a sealed glass container for a minimum of 24 h: then, when the polymer pellets were completely dissolved in the solution, the glass vials were placed in the ultrasonic bath for an additional 3 h to eliminate any bubbles that were produced during the mixing procedure.

**Figure 2:**
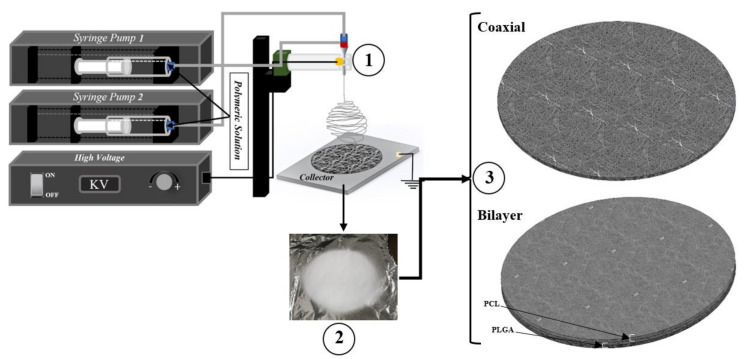
Schematics of production flat sheet electrospun coaxial and bilayer scaffolds.

The basic electrospinning set up is schematically shown in Figure 2. For the coaxial electrospinning procedure, two accurate computerised syringe pumps were necessary for dispensing both polymeric solutions. When the solutions were ready for the electrospinning procedure, solutions were drawn in the sterile Norm-JECT 20 mL syringe and mounted to the syringe pumps. However, for bilayer scaffolds, the 20G needle was used. Three different electrospun meshes with three different time intervals (30 min, 60 min and 90 min) were produced. The high voltage was adjusted each time according to the behaviour of the solution at the needle tip. The voltage was increased each time until the Tylor cone was observed. Table 1 below provides a summary of the parameters recorded during the electrospinning procedure. However, after each electrospinning procedure, the fabricated scaffolds were then placed in the vacuum chamber at room temperature for a minimum of 24 h to eliminate any remaining solvent residuals present in the membrane.

### 2.3. Scanning Electron Microscopy

Images of the scaffold surface’s morphological structure were acquired on a Hitachi TM3000 scanning electron microscope (SEM) with a 5 kV accelerating voltage. The images were taken at 1200× magnification. The average fibre diameter (µm), average pore size (µm)^2^ and scaffold surface porosity percentage were determined using SEM-assisted image analysis software. All measurements were performed using ImageJ software; this software uses a grey level on the SEM image to characterise the micrograph at the original magnification. At least 20 fibres and 20 pores were analysed for each sample image, and the average value was determined for each sample.

### 2.4. Water Contact Angle (WCA)

The wettability measurements of the electrospun coaxial and bilayer nanofibrous scaffolds were performed by static contact angle instrument (VCA-optima, AST Inc., Tacoma, WA, USA). Glass slides were used to hold the scaffolds flat for analysis. A micro-syringe was used to drop 3 µL of deionised water onto the surface of the membrane. A few seconds later, an image was captured and the contact angles were analysed and calculated. Generally, a contact angle of 90° or lower indicates better wettability (to be hydrophilic), and a contact angle above 90° signifies that the surface of the measured materials is hydrophobic.

### 2.5. Degradation Process

Two hydrolytic degradation tests were carried out in a PBS solution. The phosphate-buffered saline solution (PBS) was produced by dissolving five tablets, supplied by Fisher Scientific, USA, in 1 litre of deionised water (0.1 M, pH 7.4). The considered experiments were 12 weeks of degradation at room temperature and four weeks at controlled temperature 37 °C. All samples were cut in a rectangular shape of 5 mm × 10 mm and then submerged in 50 mL of PBS solution with 0.05% sodium azide (NaN3) to prevent microbe growth. Every week, one sample was removed from the solution and rinsed twice with distilled water to remove any minerals deposited by the PBS solution. These samples were left at room temperature in a sterilised laboratory hood overnight to evaporate any remaining liquid. When the scaffolds were dried, they were weighed and compared with the initial start date. Later, samples were further analysed under SEM to understand the scaffolds’ structural behaviour change under the degradation process condition.

### 2.6. Tensile Testing

The mechanical properties of the electrospun nanofibrous scaffolds were measured with a uniaxial testing machine (MACH-1 mechanical tester) using a single-axis 10 kg load cell under the velocity of 0.5 mm/s at room temperature condition. All samples (*n* = 3) were prepared in rectangular shape with dimensions of 35 mm × 6 mm using surgical scissors, and the thickness of each sample was measured by both digital micrometre and digital calliper. At least three samples were tested for each type of electrospun mesh.

### 2.7. Statistical Analysis

All data (at least triplicate) in this study were expressed as the mean ± standard deviations (SD). The analysis of one-way ANOVA determined statistical differences, and differences were considered statistically significant at *p* < 0.05.

## 3. Results and Discussion

The significance of this work lies primarily in its demonstration of the ability to produce porous scaffolds in which the fibre core and shell are made from two distinct polymer solutions with the help of a coaxial needle, as well as producing a bilayer membrane out of the same solution to compare their differences. Then, mainly using scanning electron microscope and appropriate image processing software, these scaffolds were characterized by morphology: fibre diameter, pore area and pore distribution. Furthermore, these scaffolds have also undergone hydrolytic degradation to observe their structural behaviour in the controlled temperature environment at 37 °C. Compared with most other studies, these results can be considered reasonable and according to the analytical methods used, the results are accurate. Compared to most other research, these results can be considered reasonable and depend on the analytical methods used and the results were accurate [44,45,46,47].

**Figure 3:**
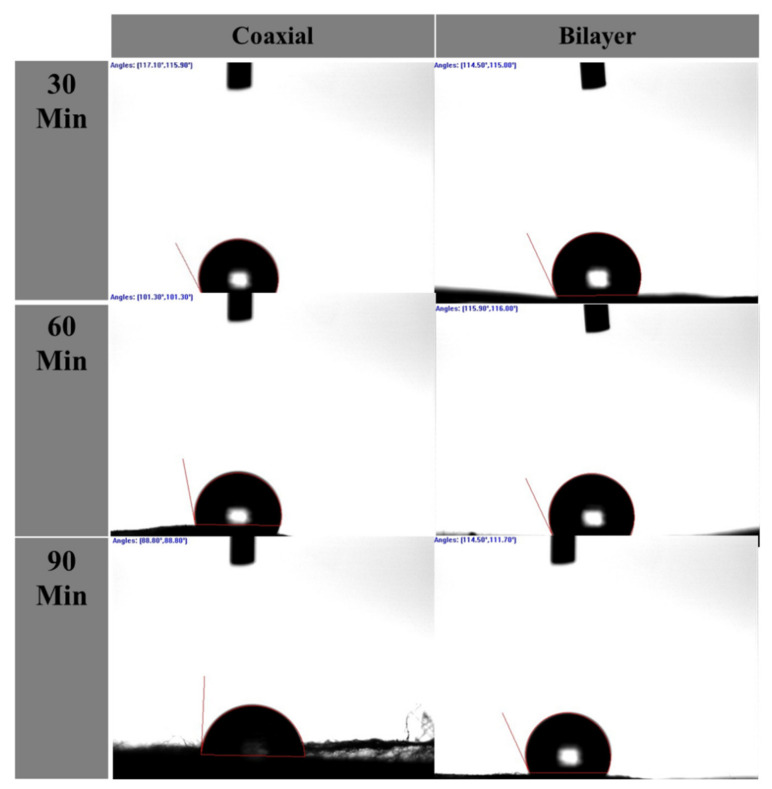
Wettability contact angle images of coaxial and bilayer scaffolds.

Studies have illustrated that tissue-engineered scaffolds’ wettability not only affects the type of protein intake, but it also has a significant impact on cell proliferation and adhesion and in-field tissue engineering and wound healing the hydrophilic membranes are more desired due to cells can attach and proliferate much more efficiently on these surfaces [48,49,50]. Since the fibre alignments and pore sizes within the porous membranes could impact the contact angle measurements, the contact angle below 90° indicated that the scaffold surface was hydrophilic and above 90° indicated that it was hydrophobic [51,52,53]. As shown in Figure 3 and Table 2 below, all electrospun porous scaffolds were shown to be very hydrophobic, which can be attributed to the nature of the PLGA and PCL polymers [54,55,56,57].

**Figure 4:**
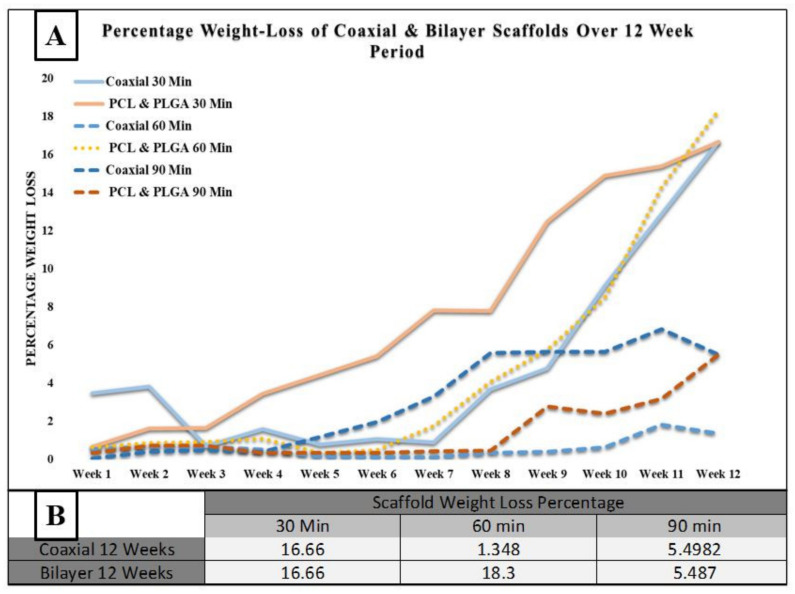
(**A**) Recorded weight loss percentage for coaxial and bilayer scaffolds over a 12-week period at room temperature; and (**B**) weight loss percentage measurements of coaxial and bilayer scaffolds over 12 weeks of degradation.

Figure 4 below shows the degradation process of the coaxial and bilayer scaffolds for 30, 60 and 90 min. One of the essential characteristics of a tissue engineering scaffold is its habitation time in the organism, mainly if it is to be used for wound healing applications. The degradation rate’s function measured the scaffolds’ weight loss percentage over the 12 weeks at room temperature. Every week the weight loss percentage of each sample was calculated and compared to the initial start date. The table in Figure 4B indicates the weight loss percentage over the degradation period. Both the 30- and 90-min scaffolds had almost the same degradation rate. However, the 60-min coaxial scaffold had significantly less weight loss than bilayer membranes, with a percentage weight loss of 1.348 and 18.3%, respectively. One of the reasons for significantly less degradation could be due to the protective layer of the shell-PLGA round the core-PCL instead of bilayer fibres.

### 3.1. Scaffold Morphology

**Figure 5:**
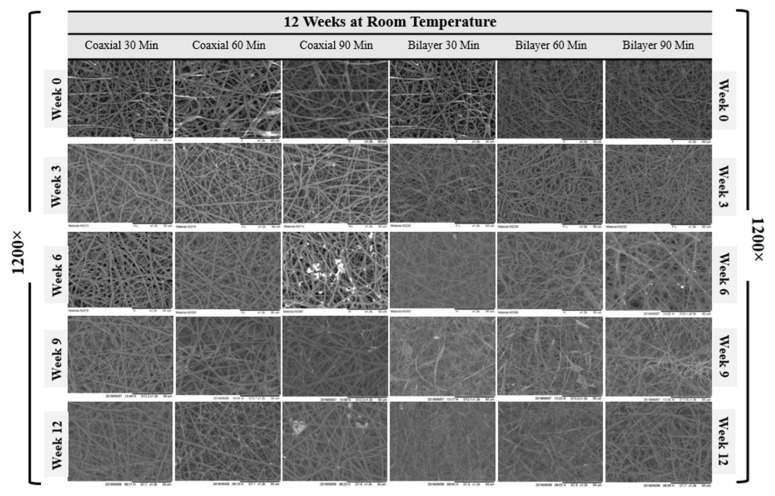
SEM images of the degradation of the coaxial and bilayer scaffolds over the 12-week period at room temperature (1200× magnification, scale bar = 50 µm).

**Figure 6:**
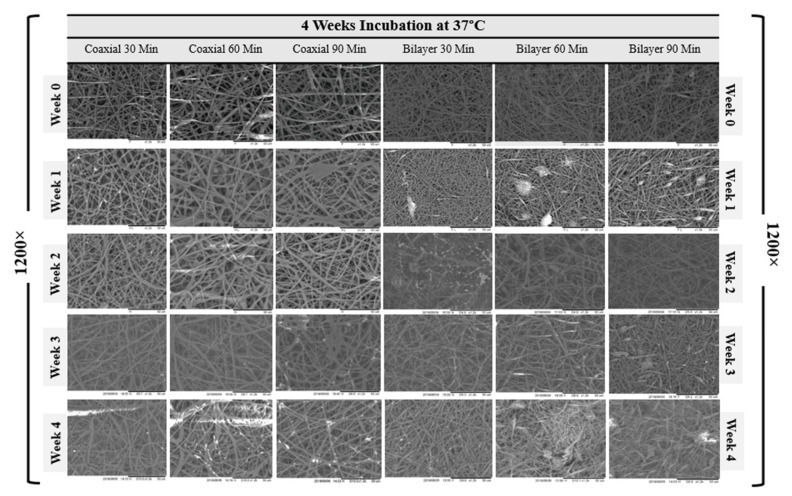
SEM images of the degradation of the electrospun coaxial and bilayer scaffolds over the 4-week period under controlled conditions (37 °C) (1200× magnification, Scale bar = 50 µm).

**Figure 7:**
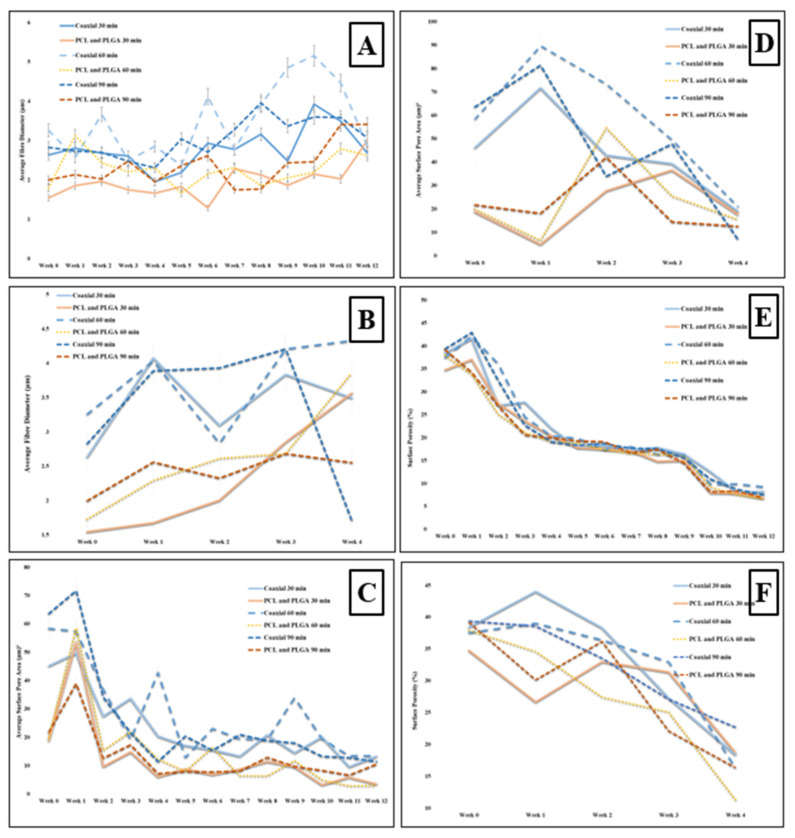
(**A**) Analysis of the change in fibre diameter (µm) of the scaffolds during the degradation period of 12 weeks, at room temperature; (**B**) analysis of the change in fibre diameter (µm) of the coaxial and bilayer scaffolds over the 4-week degradation period and under temperature-controlled conditions (37 °C); (**C**) analysis of the change in surface pore size (µm)^2^ of the electrospun coaxial and bilayer scaffolds over the 12-week degradation process at room temperature; (**D**) analysis of the change in the surface pore size (µm)^2^ of the electrospun coaxial and bilayer scaffolds over the 4-week degradation period under temperature-controlled conditions (37 °C); (**E**) analysis of the change in surface porosity in percentage of the electrospun coaxial and bilayer (PCL/PLGA) scaffolds over the degradation period of 12 weeks at room temperature; and (**F**) analysis of the change in the surface porosity percentage of the electrospun coaxial and bilayer scaffolds over the degradation period of 4 weeks under temperature controlled conditions (37 °C).

The SEM micrographs representing coaxial and bilayer membranes over 12 weeks of degradation at room temperature and four weeks at controlled 37 °C, as shown in Figure 5 and Figure 6. With the help of SEM images, it is possible to better understand the effect of degradation processes on structure, morphological changes, fibre diameter, pore size and pore distribution. Figure 7A,B show the change in mean fibre diameter of coaxial and bilayer scaffolds over 12 weeks at room temperature and four weeks at 37 °C. Both of the scaffolds displayed a well-proportioned and uniform fibre diameter with an average fibre diameter of 2.907 µm ± 0.625 SD for coaxial scaffolds and 1.758 µm ± 0.4536 SD for bilayer scaffolds. The fibre diameter seems thicker for the coaxial fibres, which again could be due to the way that the protective layer of shell-PLGA is located round the core-PCL compared to the bilayer. As early as day seven, there was an apparent change in the fibre diameters, and it was evident that polymer fibres had absorbed the moisture and the swelling of the fibres took place. The fibre diameter of the 60 min coaxial scaffold increased from 3.1 µm to 5.1 µm by week 10, and then it started to significantly decrease to 2.78 µm by the end of the 12 weeks at room temperature. As shown in Table 3A, the same coaxial scaffold showed a significant increase in its fibre diameter when it was subjected to 37 °C. However, in the case of other scaffolds, the fibre diameter measurements were different each week and showed irregularity; however, by comparing them to the coaxial scaffold, it was observed that the fibre diameter increased by the end of the 12 weeks, as well as four weeks at 37 °C for all bilayer membranes. Alongside their differences, all scaffolds have shown that they have some properties in common.

Since the scaffold’s stability is critical for cell seeding and tissue engineering operations, having scaffolds with sufficient pore size for facilitating cell migration and infiltration is extremely important—such as the tissue-engineered scaffolds made from biodegradable polymers—and observing the morphological changes, especially the pore size, during the scaffolds’ degradation at a controlled temperature (37 °C) is vital. However, before the degradation experiment, the pore size and pore distribution over the membranes’ surface were measured. The average pore size of the coaxial scaffolds was shown to be much bigger than the bilayer scaffolds, despite the fact that the volume of the pore distribution seemed to be very similar with 56.03 µm^2^ ± 17.47 SD and 38.44 % ± 1.84 SD for the coaxial scaffold and 20.16 µm^2^ ± 2.95 SD and 37.28 % ± 4.38 SD for the bilayer scaffold, respectively. This could be due to the same reason mentioned for hydrolytic degradation where the protective layer of shell-PLGA round the core-PCL provided less porosity for co-axial fibres.

The average change of surface pore size area and porosity of all electrospun coaxial and bilayer scaffolds were measured during the degradation procedures and presented in the charts below. The visible change was observed as early as the first week, and all the electrospun mats were shown to have some properties in common. As the weeks progressed, the porosity and the pore size of the scaffold significantly and steadily decreased. Despite the sharp decrease in the pore size and porosity over the degradation process, all scaffolds maintained an adequate pore size bigger than 20 µm^2^ over three weeks at 37 °C, which is a good indication which illustrates that these scaffolds are capable of allowing specific cells with a size smaller than 20 µm^2^ to penetrate and proliferate during this period in the in vitro environment.

### 3.2. Mechanical Properties

**Figure 8:**
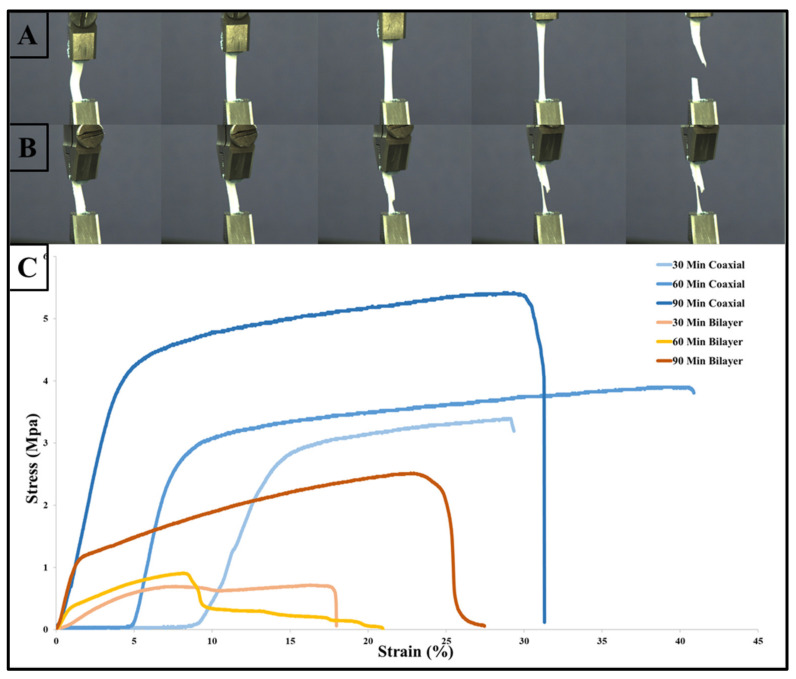
Tensile test of the electrospun coaxial and bilayer scaffolds with photographs of the flat sheet scaffold during tensile testing: (**A**) 90 min for the coaxial scaffold; (**B**) 90 min for the bilayer scaffold; and (**C**) stress–strain curve of the electrospun nanofibrous structure.

Characterization of the electrospun biodegradable membranes is meaningless unless the design of such porous membranes mimics the natural ECM of human tissues. In addition, the need to have a reliable and robust scaffold is to mechanically support cell growth and regenerate new tissues, as the mechanical properties proved to play a vital role in cell behaviour [58,59]. The changes in the polymers’ mechanical properties are usually associated with changes in their microstructures. However, when these materials are processed via electrospinning to produce fibrous scaffolds, their mechanical properties depend on the scaffolds’ structure, fibre alignments and porosity. The mechanical properties of the electrospun coaxial and bilayer scaffolds, with a process variation of 30, 60 and 90 min, were evaluated by tensile strength testing and their representative stress–strain curves are provided in Figure 8 below. The graph shows that each type of membrane has different rigidity, and by comparing these two kinds of process methods, the scaffolds produced by the coaxial needle show that they hold the highest tensile strength and elongation at break. The average Young’s modulus and tensile strength values for all coaxial and bilayer samples before the degradation are shown in Table 4 below. Although the scaffold produced by the coaxal technique was much thinner than its counterpart bilayer scaffolds, it was much stronger and more elastic than the bilayer membranes, i.e., the 60-min scaffolds fabricated the coaxial membrane with a thickness of 0.06 mm had a tensile strength of 3.87 MPa ± 1.23 SD and average elongation at break of 41.96% ± 2.48 SD compared to the bilayer scaffold which was electrospun for 60 min and had a tensile strength of 0.86 MPa ± 0.12 SD and an average elongation 21.44% ± 7.14 SD. However, the thicker coaxial scaffold exhibited smaller elongation at break. On the other hand, the bilayer membrane showed that increasing the electrospinning time could produce a more elastic membrane which will affect the tensile strength of the scaffold.

### 3.3. Handleability

**Figure 9:**
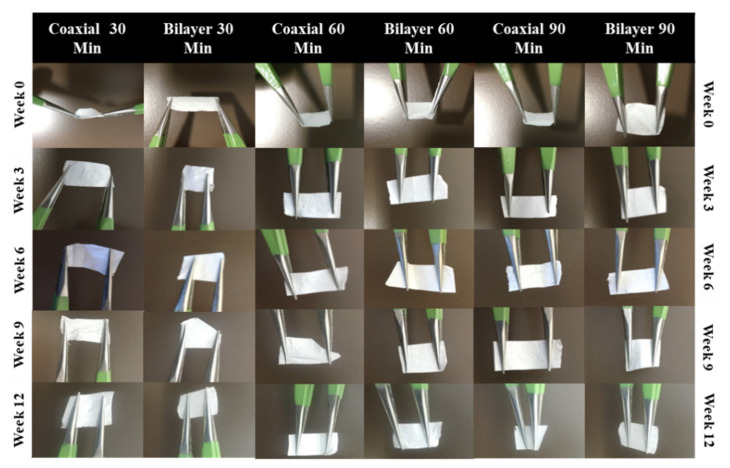
Handleability images at 90 min of the coaxial and bilayer scaffolds over the 12-week period.

From a practical point of view, the membrane’s mechanical properties should be parallel to the anatomical part of the implantation site and it should be elastic and easy to work with throughout the entire implantation or surgical process [60]. Scaffold handling is an essential factor in this study as it provides valuable information on the impact that degradation has on the scaffold’s physical behaviour. Hence, assessing the scaffolds’ handleability is a straightforward process but a vital tool to check the fabricated membranes’ functionality in various biomaterials in tissue applications. Throughout the experiment, the membranes were assessed weekly, and weeks 0, 3, 6, 9 and 12 were selected for the measurements (See Figure 9). Both the coaxial and bilayer scaffolds exhibited satisfactory handling and were transportable.

The main challenge of this work was to achieving the reproducibility of fabricated scaffolds as the electrospinning is one the most challenging techniques due to a high number of parameters affecting the produced fibres. This challenge was tackled by using a climate control chamber during the fabrication process as well as using a very high standard device.

## 4. Conclusions

In conclusion, two different electrospun porous membranes, namely coaxial (core-PCL and shell-PLGA) and bilayer scaffolds (inner layer-PCL and outer layer-PLGA), were successfully developed. The coaxial fibrous scaffolds positively changed the overall graft properties in terms of fibre diameter, pore size and mechanical properties. In terms of morphological changes during the degradation period, none of the scaffolds can be preferred over the other as they all acted differently in terms of fibre diameter changes. On the other hand, the scaffolds shrunk and encouraged the pore size and porosity of all scaffolds to significantly decrease. However, all membranes were able to retain their structural integrity over 12 weeks at room temperature, as could scaffolds which spent four weeks in the incubator at 37 °C. Overall, it can be concluded that the coaxial needle can be very advantageous in the field of tissue engineering, mainly for fabricating tubular blood vessels, which is our main aim—as will be discussed in our future publications with experiments performed by the same research team. However, the same fabricated electrospun scaffolds could have a great impact in the treatment of other tissues of the human body such as the skin, cornea, tendon and ligament.

## Figures and Tables

**Table 1:** Electrospinning process parameters that were recorded during the experiment.

	Sample Name	Voltage (kv)	Needle Type	Distance from Tip of the Needle to the Collector (mm)	Type of Collector	Flow Rate (mL/h)	T (°C)	Humidity (%)	Time (min)	Electrospinning
Coaxial	C1	13.84	Coaxial	95	Flat	Pump1: 0.5 Pump2: 0.5	21.6	43	30	0.251 each pump
	C2	14.84	Coaxial	95	Flat	Pump1: 0.5 Pump2: 0.5	22	44	60	0.532 for each pump
	C3	16.53	Coaxial	95	Flat	Pump1: 0.5 Pump2: 0.5	22.2	26	90	0.751 for each pump
PCL and PLGA	D1	Voltage for PCL: 7.55 Voltage for PLGA: 7.05	20G	95	Flat	1	21.2	28	15 min for PCL 15 min for PGA	PCL: 0.278 mL PLGA: 0.273 mL
	D2	Voltage for PCL: 7.52 Voltage for PLGA: 7.00	20G	95	Flat	1	21.2	28	30 min for PCL and 30 min for PLGA	PCL: 0.543 mL PLGA: 0.545 mL
	D3	Voltage for PCL: 7.52 Voltage for PLGA: 7.11	20G	95	Flat	1	21.2	28	45 min for PCL and 45 min for PLGA	PCL: 0.756 mL PLGA: 0.752 mL

**Table 2:** Mean ± SEM of contact angle measurements of coaxial and bilayer (PCL and PLGA) scaffolds.

Scaffold with Spinning Period (min)		Mean ± SD	(DH2O)
		Left Angle	Right Angle
Coaxial	30 min (C1)	122.8° ± 12.3	122.7° ± 13.8
	60 min (C2)	110.42° ± 21.5	107.42° ± 19.3
	90 min (C3)	106.42° ± 35.9	106.2° ± 33.4
PCL and PLGA	30 min (D1)	120.37° ± 22.5	120.65° ± 23.3
	60 min (D2)	120.45° ± 13	120.8° ± 13.3
	90 min (D3)	116.72° ± 17.5	115.45° ± 16.5

**Table 3:** (**A**) Percentage change in fibre diameter for the coaxial and bilayer scaffolds electrospun for 30, 60 and 90 min; (**B**) percentage change in surface pore size for the coaxial and bilayer scaffolds electrospun for 30, 60 and 90 min; and (**C**) percentage change in surface porosity percentage for the coaxial and bilayer scaffolds electrospun for 30, 60 and 90 min. Red arrow = decrease and blue arrow = increase.

**A**	**Percentage Change in Fibre Diameter**		
	30 min	60 min	90 min
Coaxial 12 Weeks	2.18 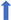	11.97 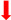	8.35 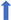
Bilayer 12 Weeks	93.61 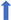	46.01 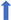	70.68 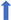
Coaxial 4 Weeks at 37 °C	32.69 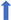	32.82 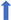	39.541 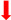
Bilayer 4 Weeks at 37 °C	130.61 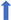	123.23 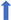	27.61 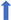
**B**	**Percentage Change in Pore Size**		
	30 min	60 min	90 min
Coaxial 12 Weeks	70.15 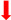	77.00 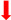	82.04 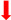
Bilayer 12 Weeks	81.60 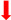	85.43 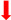	51.53 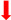
Coaxial 4 Weeks at 37 °C	58.69 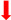	64.03 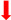	88.87 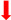
Bilayer 4 Weeks at 37 °C	5.75 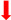	22.27 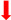	42.47 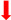
**C**	**Percentage Change in Surface Porosity (%)**		
	30 min	60 min	90 min
Coaxial 12 Weeks	78.57 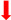	75.35 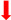	80.75 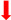
Bilayer 12 Weeks	80.33 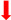	82.53 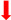	82.4 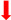
Coaxial 4 Weeks at 37 °C	52.10 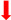	56.49 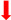	42.25 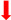
Bilayer 4 Weeks at 37 °C	46.08 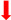	70.03 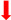	58.17 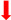

**Table 4:** Summary of the mechanical properties of the coaxial and bilayer scaffolds spun for 30, 60 and 90 min.

Sample Name	Time	Length (mm)	Thickness (mm)	Width (mm)	Area (mm)^2^	Tensile Strength (MPa ± SD)	Elongation at Break (% ± SD)	Young Modulus (MPa ± SD)
Coaxial	30	35.6	0.04	5	0.2	3.29 ± 1.65	29.16 ± 4.02	21.74 ± 3.45
	60	34	0.06	5.5	0.33	3.87 ± 1.23	41.96 ± 2.48	29.44 ± 7.14
	90	35.3	0.075	6.2	0.465	5.39 ± 1.64	31.23 ± 6.74	33.84 ± 4.21
Bilayer	30	26.8	0.17	5.3	0.901	0.68 ± 0.1	17.86 ± 3.11	3.77 ± 1.53
	60	26	0.18	5.5	0.99	0.86 ± 0.12	21.44 ± 7.14	4.31 ± 1.22
	90	27.1	0.21	4.52	0.949	2.94 ± 0.52	22.35 ± 3.64	7.40 ± 2.15

## Data Availability

Not applicable.
